# Coronavirus and Obesity: Could Insulin Resistance Mediate the Severity of Covid-19 Infection?

**DOI:** 10.3389/fpubh.2020.00184

**Published:** 2020-05-12

**Authors:** Francis M. Finucane, Colin Davenport

**Affiliations:** ^1^HRB Clinical Research Facility, National University of Ireland Galway, Galway, Ireland; ^2^Bariatric Medicine Service, Centre for Diabetes, Endocrinology and Metabolism, Galway University Hospitals, Galway, Ireland

**Keywords:** COVID 19, insulin resistance, ACE2, obesity, coronavirus

## Introduction

The rapidly evolving global epidemic of coronavirus disease 2019 (COVID-19) (1), caused by the novel severe acute respiratory syndrome corona virus 2 (SARS-CoV-2), presents with variable clinical severity, being fatal in some and asymptomatic in others (2). Preliminary indications from the UK (3), China (4), and the USA (5) suggest that patients with obesity, at least in hospital, have a worse prognosis. This is consistent with long-established observations that patients with acute respiratory distress syndrome (ARDS) who have obesity do worse, for several reasons (6). Obesity causes atelectasis, particularly in the posterior dependant lung zones (7) and this, along with collapse of alveolar capillary units because of raised pleural pressures (8) leads to diminished recruitability of lung tissue. Also, parenchymal heterogeneity leads to high lung shear forces, even when applied ventilatory pressures are low and without well-established lung injury (9). This is consistent with population-based observations that obesity adversely affects lung function (10).

However, it is becoming increasingly apparent that there may be more specific mechanisms by which obesity worsens the outcome of COVID-19, arising from metabolic and inflammatory derangements rather than merely the mechanical effects of increased fat tissue. For example, categorizing overweight and obesity in the recent audit of intensive care patients with Covid-19 (who had a mean age of 60 years) (3), their prevalence was very similar to that in the background population of older British adults (11). In a case series of 112 Chinese adults with prevalent cardiovascular disease and Covid-19, 88% of those who died vs. 19% of those who survived were categorized as overweight or obese, but only 16 patients in this small study received intensive care and the mean body mass index (BMI) was 22 vs. 25.5 kgm^−2^ for normal weight and overweight patients, respectively (4). Thus, there may have been substantial differences in the proportions of patients who died in the normal weight vs. overweight categories, without there being a strong or clinically relevant association between BMI and mortality, especially when relevant confounders are not taken into consideration. A larger French study showed that COVID-19 patients with severe obesity in intensive care were seven times more likely to require invasive mechanical ventilation compared to Covid-19 intensive care patients with a normal BMI, but this trend was not statistically significant for those with BMI <35 kg m^−2^ (12). This is similar to observations from large prospective cohort studies of hospitalized patients during the 2009 Influenza (H1N1) pandemic, which showed that severe obesity was associated with a much higher risk of intensive care admission and mortality, but overweight and obesity were not (13). Despite an exhaustive literature search, we were unable to identify any studies that found BMI (as a continuous variable) to be a good predictor of outcomes in patients with SARS or COVID-19.

Conversely, several of the features of the so-called metabolic syndrome seem to be associated with a worse prognosis in patients with COVID-19. Males seem to be worse affected than females, with a male preponderance in several studies (3, 14–16). Older Covid-19 patients were found to have a worse prognosis in China (2, 16), Italy (17) and the UK (3). As was seen with SARS in 2003 (18), diabetes and dysglycaemia have been found to be highly prevalent in Covid-19 patients (16, 19). They often have a transaminitis (16, 19), usually attributed to shock but which might indicate underlying fatty liver disease. Hypertension is associated with worse outcomes (17, 20). These early clinical observations that patients with severe Covid-19 tend to be older, male, hypertensive, with elevated blood glucose levels and abnormal liver blood tests raise the prospect that insulin resistance could play an important role in mediating disease severity. Is there a plausible mechanistic theory for such an association?

## Mechanisms Linking Insulin Resistance and Covid-19 Severity

Insulin resistance arises from defective insulin action in its target tissues—primarily skeletal muscle, liver and white adipose tissue—either as a result of insulin receptor defects or much more commonly due to perturbations in the post-receptor insulin signaling cascade (21). While several factors such as exercise, oxidative stress and inflammation modulate insulin action, the pathological levels of insulin resistance associated with metabolic disease are driven by chronic overnutrition and ectopic fat accumulation in target tissues. Thus, at normal plasma insulin concentrations, these tissues are unable to mount a co-ordinated physiological response to lower glucose through suppression of endogenous glucose production in the liver and glucose uptake and glycogen synthesis in muscle. As a result, impaired insulin action is associated with increased circulating insulin concentrations (22).

Angiotensin Converting Enzyme 2 (ACE2) is a potentially important molecular link between insulin resistance and COVID-19 severity. It serves as the ligand through which coronaviruses like SARS-CoV-2 bind to their target cells (23). ACE2 is expressed in numerous tissues including lung alveolar epithelial cells, pancreatic beta cells and enterocytes of the small intestine (24). The main physiological role of ACE2 is the conversion of angiotensin 2, a vasoconstricting, profibrotic and proinflammatory molecule into angiotensin 1–7, a vasodilator (25). Crucially, angiotensin 2 is the predominant component of the renin-angiotensin-aldosterone system (RAAS) that drives insulin resistance and cardiovascular dysfunction (26, 27). By degrading Angiotensin 2, ACE2 protects against the effects of RAAS overactivation, reducing insulin resistance by decreasing cellular oxidative stress, enhancing insulin signaling and insulin-stimulated glucose transport activity (28).

Given its critical protective role, it is not surprising that several mechanistic studies (29) have confirmed that ACE2 expression is increased in rodents fed a high sucrose diet (30) or given insulin sensitisers (31). The effects of insulin on ACE2 expression are tissue specific, with reduced expression in NOD mouse glomerular podocytes (32) but increased ACE2 expression in NOD mouse lungs after insulin administration (33). A recent very large “phenome-wide” Mendelian Randomization study by Rao et al. has just confirmed that several diabetes-related traits are associated with increased lung ACE2 expression (34). Muniyappa and colleagues have astutely proposed that this might mediate the association between diabetes and COVID-19 severity, but hypothesized that elevated glucose rather than elevated insulin levels were the underlying metabolic driver of increased ACE2 expression (35). However, of note in the Rao study was the finding that insulin therapy was independently associated with lung ACE2 expression (34). This distinction might have clinical relevance as it would determine whether to prioritize the normalization of blood glucose vs. insulin levels, in order to reduce ACE2 expression and ultimately COVID-19 severity.

It is clear also that other mechanisms, independent of ACE2 expression, are likely to contribute to the more severe phenotype associated with diabetes in COVID-19 (36). A “cytokine storm” has been implicated in the multi-organ failure associated with Covid-19 and there is good evidence from animal models of Middle-East Respiratory Syndrome (MERS) that diabetes alters the cytokine profile and aggravates a dysregulated immune response which worsens lung pathology (37). Also the observations that elevated plasma glucose levels and diabetes are independent risk factors for mortality and morbidity in patients with SARS (18) and COVID-19 (16, 19) are beyond doubt, but consideration needs to made that these could reflect, at least in part, a state of insulin resistance and elevated insulin levels that are driving increased ACE2 expression in lung epithelial cells and aggravating disease severity.

## Implications of Potential Role of Insulin Resistance in Covid-19 Severity

While most clinicians are aware of the concept of insulin resistance, it is never measured in routine clinical practice and is at most an abstract, intangible and academic consideration. Even experienced clinical experts in endocrinology and related specialties tend to simply dichotomise patients as either being insulin resistant or not. This makes it difficult to determine the influence of insulin resistance on patient outcomes in the preliminary Covid-19 studies that have been published to date, as it hasn't been a consideration in the clinical characterization of these patients and the data simply aren't available. This makes it difficult to determine its utility in predicting COVID-19 severity, response to interventions or therapeutic trajectories, or to assess its relative importance compared to hypertension, diabetes or obesity. It would be premature then to suggest that measuring insulin resistance should form part of routine clinical assessment of these patients. However, it seems reasonable to explore the potential of the insulin resistant phenotype as a prognostic indicator, as well as determining whether changes in insulin sensitivity during COVID-19 infection are associated with altered outcomes.

There are a number of ways to assess insulin resistance. While the gold-standard hyperinsulinaemic-euglycaemic clamp technique allows precise quantification of hepatic and skeletal muscle insulin sensitivity (38), it is technically demanding, time consuming and expensive and we do not think it would be feasible in most centers. The leptin: adiponectin ratio (LAR) has been validated as a robust measure of whole body insulin sensitivity in large epidemiological studies (39, 40). Both of these molecules are adipokines, secreted exclusively by adipocytes and are important regulators of metabolic homeostasis. Leptin acts on the hypothalamus to regulate food intake and energy expenditure (41) with obese individuals having higher leptin levels (42). Conversely adiponectin increases tissue fat oxidation and reduces circulating free fatty acids and is lower in obese individuals (43). Other methods based on fasting insulin and glucose levels (44) and the dynamic response to oral glucose loading (45) may be less feasible in the acutely unwell patient. Clinical signs of insulin resistance such as acanthosis nigricans, androgenetic alopecia, acne or hirsutism (46–48) could also be considered. We (49) and others (50, 51) have found that acrochordons (skin tags) are associated with dysglycaemia and hypertension in patients with obesity and are a useful marker of insulin resistance in patients presenting with atypical diabetes phenotypes (52, 53).

We think that a prospective cohort study which measures the clinical (skin tags, acanthosis nigricans, waist: hip ratio) and biochemical (leptin, adiponectin, fasting insulin, fasting glucose) variables associated with insulin resistance, in order to determine if they were associated with COVID-19 severity seems warranted. This could be conducted with a relatively low participant burden in patients admitted to hospital. Of potentially more relevance and utility in identifying those at risk from COVID-19 would be to examine the large, well-established prospective epidemiological cohort studies which have focused on precise measurement of insulin sensitivity [for example the EGIR RISC study (54)] and determine whether this predicts COVID-19 incidence or severity. Alternatively, large cohorts of prospectively genotyped patients such as the UK Biobank (55) could identify genetic polymorphisms associated with COVID-19 incidence or severity that would enhance our understanding of the mechanistic basis for the variation in severity of the infection. These studies, unlike those in cohorts of patients recruited during acute COVID-19 infection, would have the advantage of excluding reverse causality as the basis for any observed association between insulin resistance and severity of infection: Coronavirus infections are known to cause diabetes (56) and severe inflammation in itself can worsen insulin resistance. Finally, if an association between insulin resistance and COVID-19 severity was established, the next step would be to determine whether strategies to enhance insulin sensitivity acutely (such as carbohydrate restriction) could improve prognosis.

## Conclusions

The variable severity of COVID-19 infection is likely to be multifactorial, and age, sex, severe obesity and diabetes are well-established risk factors for increased morbidity and mortality. However, the extent to which insulin resistance contributes to these associations is not known and may be substantial, especially given the critical role of the ACE2 ligand in determining disease severity. Therefore, clinical and biochemical markers of insulin resistance should be evaluated for their prognostic utility. Furthermore, if an association between insulin sensitivity and COVID-19 severity is found, consideration should be given to assessing therapeutic interventions to enhance insulin sensitivity and improve outcomes.

## Author Contributions

FF and CD conceptualized the paper, conducted the literature review, drafted and revised the manuscript, and approved the final version.

## Conflict of Interest

In the past (up to 2017), FF received honoraria, travel grants and served on advisory boards for Novo Nordisk, Eli Lilly, Pfizer Inc., Sanofi-Aventis, Astra Zeneca, Merck-Serono, Boehringer Ingelheim, Janssen and Novartis. The remaining author declares that the research was conducted in the absence of any commercial or financial relationships that could be construed as a potential conflict of interest.
